# Selective repression of retinoic acid target genes by RIP140 during induced tumor cell differentiation of pluripotent human embryonal carcinoma cells

**DOI:** 10.1186/1476-4598-6-57

**Published:** 2007-09-19

**Authors:** Kelly C Heim, Kristina A White, Dexin Deng, Craig R Tomlinson, Jason H Moore, Sarah J Freemantle, Michael J Spinella

**Affiliations:** 1 Department of Pharmacology and Toxicology, Dartmouth Medical School, Hanover, USA; 2 Department of Genetics, Dartmouth Medical School, Hanover, USA; 3 Norris Cotton Cancer Center, Dartmouth Hitchcock Medical Center, Lebanon, USA

## Abstract

**Background:**

The use of retinoids as anti-cancer agents has been limited due to resistance and low efficacy. The dynamics of nuclear receptor coregulation are incompletely understood. Cell-and context-specific activities of nuclear receptors may be in part due to distinct coregulator complexes recruited to distinct subsets of target genes. RIP140 (also called NRIP1) is a ligand-dependent corepressor that is inducible with retinoic acid (RA). We had previously shown that RIP140 limits RA induced tumor cell differentiation of embryonal carcinoma; the pluriopotent stem cells of testicular germ cell tumors. This implies that RIP140 represses key genes required for RA-mediated tumor cell differentiation. Identification of these genes would be of considerable interest.

**Results:**

To begin to address this issue, microarray technology was employed to elucidate in a *de novo *fashion the global role of RIP140 in RA target gene regulation of embryonal carcinoma. Subclasses of genes were affected by RIP140 in distinct manners.

Interestingly, approximately half of the RA-dependent genes were unaffected by RIP140. Hence, RIP140 appears to discriminate between different classes of RA target genes. In general, RIP140-dependent gene expression was consistent with RIP140 functioning to limit RA signaling and tumor cell differentiation. Few if any genes were regulated in a manner to support a role for RIP140 in "active repression". We also demonstrated that RIP140 silencing sensitizes embryonal carcinoma cells to low doses of RA.

**Conclusion:**

Together the data demonstrates that RIP140 has profound effects on RA-mediated gene expression in this cancer stem cell model. The RIP140-dependent RA target genes identified here may be particularly important in mediating RA-induced tumor cell differentiation and the findings suggest that RIP140 may be an attractive target to sensitize tumor cells to retinoid-based differentiation therapy. We discuss these data in the context of proposed models of RIP140-mediated repression.

## Background

Retinoids are used in the clinic to prevent and treat select tumors through the process of induced tumor cell differentiation, yet the efficacy of retinoids toward the most common neoplasms has been limited [1]. Therefore, it is important to understand the mechanisms by which tumors are resistant to the ability of retinoids to affect tumor growth and progression. Embryonal carcinoma (EC) is a model of embryonic development as well as tumor cell differentiation [2]. EC is considered to be the malignant counterpart to embryonic stem cells because of a remarkable degree of genetic, morphologic, biochemical and gene expression similarity [3,4]. EC cells are pluripotent and considered to be the stem cells of male germ cell tumors [3,4]. In response to all *trans *retinoic acid (RA), the pluripotent human EC cell line, NT2/D1, differentiates toward a neuronal lineage with associated loss of cell growth and tumorigenicity [5,6].

Retinoids mediate their effects by binding to and activating retinoid specific nuclear receptors, RARs and RXRs [7,8]. Corepressors, such as NCoR and SMRT, bind to nuclear receptors in the absence of ligand to assemble large repressor complexes containing chromatin repressing and histone deacetylase activity [9,10]. Ligand-bound receptors recruit coactivators, such as the p160 family, which recruit histone acetyltransferases and other chromatin remodeling proteins to induce gene transcription [11].

This classic coregulator paradigm does not fully explain the recent evidence that ligands such as retinoids, estrogen, and vitamin D3, apparently also actively suppress gene expression [12,13]. These data challenge the current coregulator paradigm by implying that repressor complexes may participate in active repression of ligand-bound receptors. RIP140 is the first member of a unique class of ligand-dependent corepressors [14-18]. RIP140 exhibits intrinsic repression activity when fused to the Gal4-DNA binding domain, and directly recruits histone deacetylases as well as the transcriptional repressor, c-terminal binding protein (CTBP), to nuclear receptors [19,20]. In addition, RIP140 inhibits the transactivation function of several ligand bound nuclear receptors [14-18]. Since RIP140 preferentially binds and suppresses agonist bound nuclear receptors, it provides a candidate mechanism for active repression of several members of the nuclear receptor superfamily.

Our prior work demonstrated that RIP140 is a direct target of retinoid signaling and that RIP140 can be limiting for both retinoid and estrogen signaling [21-23]. Silencing RIP140 in NT2/D1 cells resulted in RA-dependent increases in expression of model endogenous RA target genes RARβ and LEFTY2, and enhanced and accelerated RA-induced differentiation and growth suppression [22]. This evidence suggests that RA induction of RIP140 constitutes a negative-feedback mechanism to fine-tune retinoid signaling.

Retinoids are pleiotropic agents regulating many gene targets. However, we have shown that RIP140 siRNA accelerates RA-dependent growth suppression and differentiation [22]. The identification of genes normally limited by RIP140 during RA-induced growth and differentiation is of interest since these genes may be particularly important regulators of these processes. The possibility remains that unidentified subclasses of genes may be regulated by RIP140 in distinct manners [22].

The present study was conducted to characterize, on a genome-wide scale, the role of RIP140 in the regulation of RA signaling in a cancer stem cell model. Together the data demonstrates that RIP140 has profound effects on RA-mediated gene expression. Subclasses of genes were affected by RIP140 in distinct manners while other RA-dependent genes were unaffected suggesting that RIP140 may discriminate between different classes of RA targets. The RIP140-dependent RA target genes identified here may be particularly important in mediating RA-induced tumor cell differentiation.

## Results

### Validation of RIP140 specific siRNA

We previously established that RIP140 is a target of RA signaling and that RIP140 in turn is a limiting factor for RA activity [22]. Specifically, we showed that knockdown of RIP140 with a RIP140-specific siRNA (RIP140siRNA#1) increased endogenous expression of RA target genes RARβ and LEFTY2, and accelerated immunophenotypic differentiation as measure by A2B5 staining of NT2/D1 cells [22]. A key feature was that knockdown of RIP140 did not affect these endpoints in cells depleted of endogenous retinoids, confirming that RIP140 mainly associates with ligand-bound nuclear receptors. These studies were performed with a constant 1 μM dose of RA and a relatively high, 150 nM dose of siRNA. Prior to global gene expression studies, we wished to further validate the siRNA approach for targeting RIP140.

**Figure 1:**
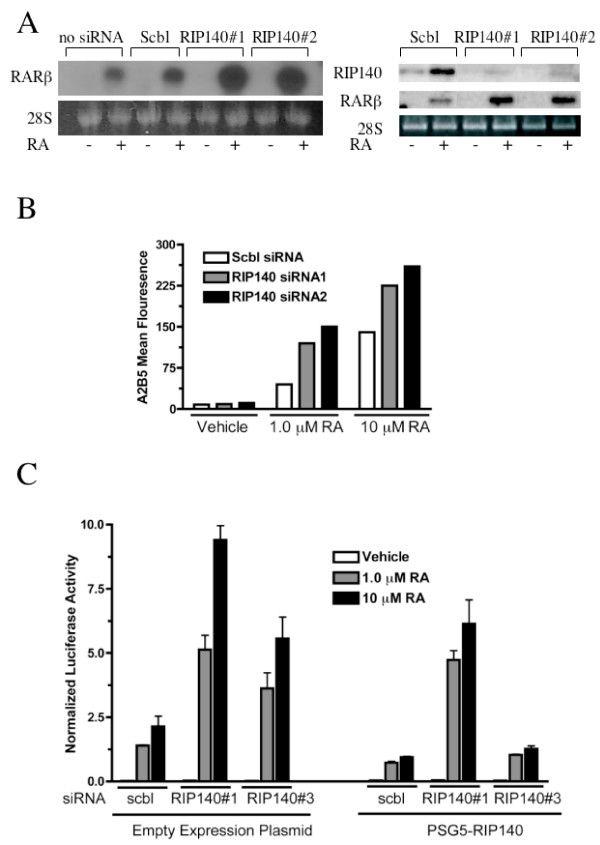
**RIP140 silencing enhances ligand-dependent RAR activation and tumor cell differentiation**. **(A, left) **Northern analysis depicting the effect of RIP140 siRNA on expression of known retinoic acid receptor target gene RARβ in NT2/D1 cells. Cells were mock transfected (no siRNA) or transfected with 150 nM siRNA prior to treatment with 1 μM RA for 24 hours. **(A, right) **Northern analysis depicting the effect of RIP140 siRNA on expression of RARβ. Cells were transfected with 40 nM siRNA prior to treatment with 1 μM RA for 24 hours. Scbl, scrambled. **B) **Immunophenotypic FACS analysis for the established neuroectodermal marker A2B5 in NT2/D1 cells transfected with 40 nM scrambled siRNA (Scbl) or RIP140 siRNA#1 or RIP140 siRNA#2 and treated for 4 days with DMSO vehicle control or indicated dosages of RA. Representative of two experiments. **(C) **The effect of RIP140 siRNAs and exogenous RIP140 expression on activity of an RARE-TK-Luc promoter in NT2/D1 cells. Cells were transfected with indicated control (scbl) or RIP140 siRNAs at a concentration of 50 nM and either empty expression vector or a plasmid expressing RIP140 prior to addition of indicated dosages of RA for 48 hours. Note, RIP140 siRNA#1 targets sequences within the RIP140 expression plasmid while RIP140 siRNA#3 targets the 3' untranslated region of RIP140 but does not target PSG5-RIP140. Error bars are S.D. of triplicate determinations. For all experiments above, cells were cultured in charcoal absorbed sera media for 24 hours prior to and during the experiment.

Experiments were performed under charcoal absorbed sera conditions to deplete the basal levels of retinoids known to be in sera. An additional siRNA, RIP140siRNA#2, also potently enhanced the RA-mediated expression of the RA target gene RARβ at 150 nM and at doses as low as 40 nM (Figure 1A). Importantly, RIP140 siRNA cells also overexpressed RARβ when compared to mock transfected cells (Figure 1A). Low, 40 nM doses of RIP140siRNA#1 and RIP140siRNA#2 resulted in enhanced neuronal differentiation with RA as assessed by expression of the early neuronal ectodermal marker, A2B5 (Figure 1B).

An important control to ensure a given siRNA phenotype is not due to off-target effects is to rescue the siRNA phenotype with overexpression of its target. RIP140siRNA#1, as well as a newly designed siRNA targeting the 3' untranslated region of human RIP140 (siRNA#3) enhanced induction of a RAR-specific reporter construct in a RA-dependent manner (Figure 1C). Further, a RIP140 expression plasmid that does not contain the 3' untranslated region of RIP140 repressed RA signaling in scrambled control cells (Figure 1C). This RIP140 construct potently opposed the effect of RIP140siRNA#3 but not RIP140siRNA#1, which targets the coding region of RIP140. Thus, silencing of RIP140 by siRNA enhanced RA-mediated responses in NT2/D1 cells at low doses of siRNA and this could be rescued by exogenous RIP140. Further, the effects of RIP140 silencing were mainly ligand-dependent since performing these experiments under charcoal absorbed sera conditions resulted in minimal changes in basal RAR activity or in the differentiation of NT2/D1 cells (Figure 1).

### Microarray Design

**Figure 2:**
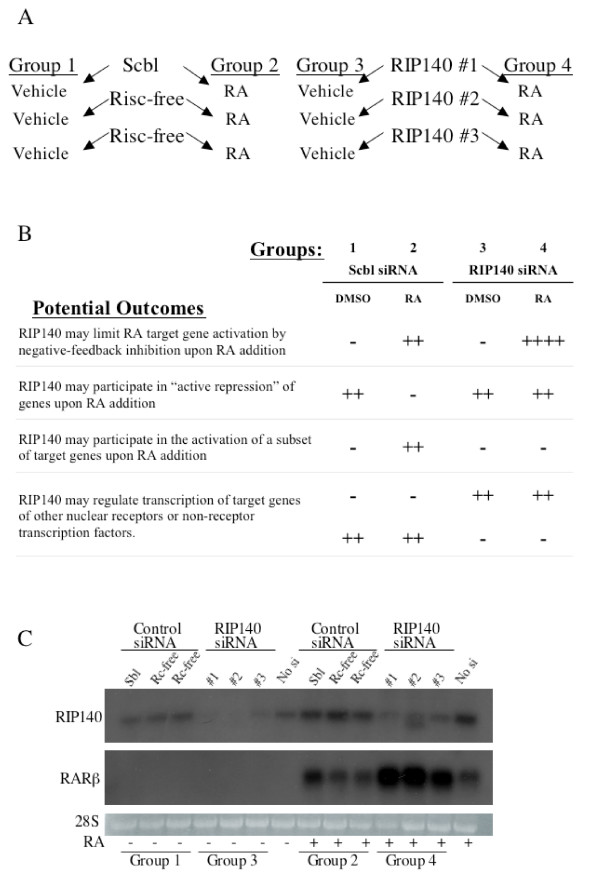
**Microarray design**. **(A) **Cells cultured in normal sera media were transfected with 90 nM control siRNAs (scrambled or RISC-free) or three separate RIP140 siRNAs. Cells were than split into DMSO, vehicle treatment or 10 μM RA treatment for 24 hours prior to harvesting for mRNA. **(B) **Possible outcomes of RIP140 siRNA on RA-dependent and RA-independent gene expression. -, no change or no expression; ++ or ++++, expression or increased expression. **(C) **Northern analysis of actual samples for microarray analysis depicting repression of RIP140 expression with indicated RIP140 siRNA and associated enhancement of RARβ expression. Included are mock transfected cells (no siRNA).

We then sought to determine the global effects of RIP140 on RA-dependent and RA-independent gene expression. A dose of siRNA of 90 nM was chosen. Twelve independent hybridizations were performed from mRNA isolated from 12 independent samples (Figure 2A). One scrambled and two independent RISC-free siRNA transfections and transfections with three distinct RIP140 siRNAs were performed in NT2/D1 cells. Each siRNA treatment was further treated with either 10 μM RA or DMSO control for 24 hours. Hence, the design consisted of four groups, the three siRNA controls treated with vehicle, Group 1; the siRNA controls treated with RA, Group 2; siRNA to RIP140 treated with vehicle, Group 3; and siRNA to RIP140 treated with RA, Group 4 (Figure 2A).

The treatments were done in the presence of normal sera, since charcoal absorption of sera removes many small hydrophobic ligands to the nuclear receptor family in addition to retinoids. This measure was taken with the aim to perhaps identify target genes of other nuclear receptors including orphan receptors that could be regulated by RIP140 in the presence of low levels of their cognate ligands. RA treatment of 10 μM for 24 hours was chosen since previous experience with NT2/D1 cells indicated that a reasonably sized set of altered genes would be obtained [5,24]. In addition, NT2/D1 cells commit to RA-induced differentiation by 48 hours and RIP140 repression accelerates RA-induced differentiation of EC cells [5,22]. Therefore, the 24-hour time point is well-within the range of the RA commitment "window" and a larger percentage of direct RA-target genes could be expected to be uncovered compared to later time points.

Based on the current understanding of the properties of RIP140 [14-18], a number of potential outcomes were anticipated: 1) RIP140 may limit RA target gene activation by negative-feedback inhibition upon RA addition, 2) RIP140 may participate in "active repression" of gene expression upon RA addition, 3) an unexpected outcome that RIP140 may participate in the activation of a subset of target genes, 4) RIP140 may regulate the transcription of target genes of other nuclear receptors or even non-nuclear receptor transcription factors. The readout from the array design that would support each of these outcomes is depicted in Figure 2B. The extent of RIP140 knockdown and the level of enhancement of RARβ expression in the actual samples used in for array are depicted in Figure 2C.

### Silencing of RIP140 quantitatively alters the RA transcriptional response in EC cells

**Figure 3:**
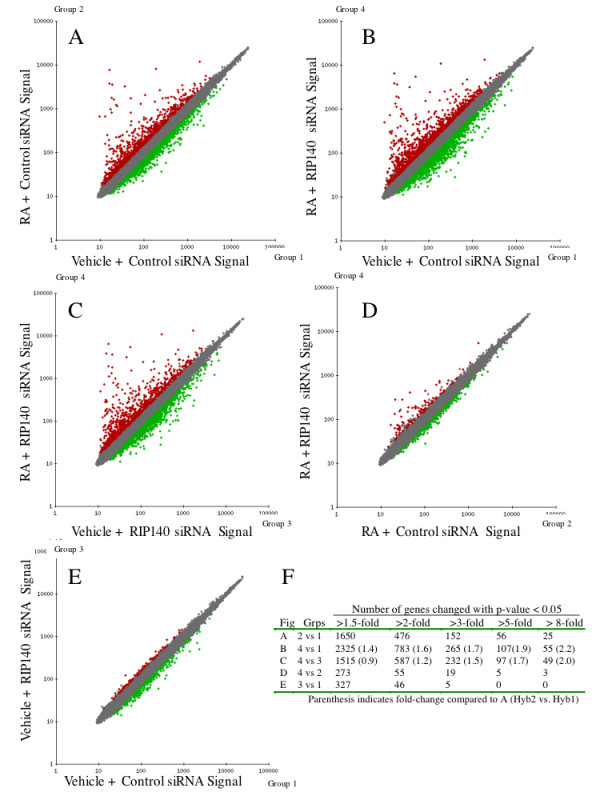
**Scatter plot analysis of gene expression differences between groups**. **(AE) **The three independent samples for each of the four groups as outlined in Figure 2A were averaged and then compared to one another utilizing the GeneSifter package as described in Materials and Methods. Red squares represent genes expressed to a higher degree in samples along the y-axis; green squares represent genes expressed to a higher degree in samples along the x-axis. Grey squares represent genes changed less than 1.5 fold or with a p-value that failed to reach 0.05. **(F) **Table depicting the number of genes in the pairwise comparisons above (A-E) that are altered above the fold-cut offs indicated and with a p-value < 0.05.

To further ensure that off-target effects were minimized, any gene that differed 1.5-fold or greater between the replicates of the control (Group 1) were removed from the analysis. The expression of the remaining 52,270 probe sets were compared in pairwise fashion between the experimental groups (Figure 3). As expected, comparison of gene expression between the siRNA control cells treated with RA and the control cells treated with vehicle revealed that the great majority of genes are unaltered with RA treatment (Figure 3A). Interestingly, the total number of genes regulated by RA in the RIP140 siRNA cells increased compared to the number of genes regulated by RA in the siRNA control cells (compare the spread in the scatter plots between Figure 3A vs. Figure 3B or 3C). This is apparent regardless of whether RIP140 siRNA RA cells were compared with control siRNA vehicle cells (Figure 3B) or compared to RIP140 siRNA vehicle cells (Figure 3C). The disparity in the overall number of RA-sensitive genes increased as the fold-cut off increased (Figure 3F), suggesting that RIP140 siRNA increases the number of RA altered genes and also amplifies RA-dependent gene alterations. It is important to emphasize that the genes identified here under these experimental conditions may not be direct target genes of liganded RARs. The complete list of genes and expression values for each pairwise comparison is supplied in Supplemental Tables S1 to S5 [see Additional file 1].

**Figure 4:**
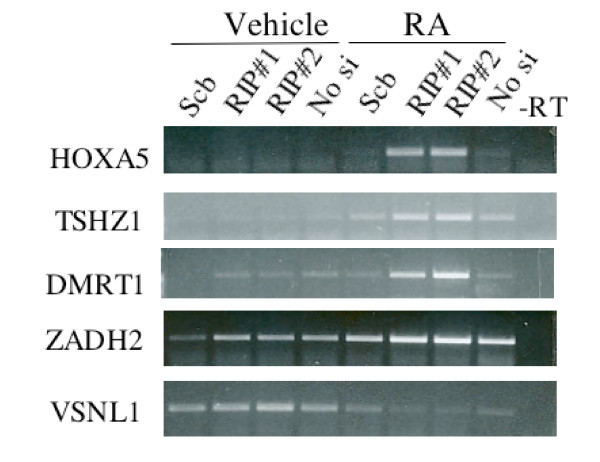
**Genes differentially expressed with RA in RIP140 siRNA treated NT2/D1 cells**. Five of top six genes in which RA-regulation was altered in RIP140 siRNA cells (Table 1) were confirmed by RT-PCR analysis. Samples used were the same as those used in the microarray analysis (Figure 2C). Cells were mock transfected (no siRNA) or transfected with RIP140 siRNA#1 or RIP140 siRNA#2 prior to treatment with vehicle or 10 μM RA for 24 hours. In indicated samples (-RT) reverse transcriptase was omitted to control for genomic DNA contamination.

A similar trend is also apparent in the scatter plot comparing RIP140 siRNA RA cells with control siRNA RA cells (Group 4 vs. Group 2, Figure 3D). The greatest difference is in RA induced genes, consistent with a role for RIP140 in repressing the activation of ligand engaged RARs. However, a number of repressed genes are also repressed to a greater extend in RIP140 siRNA cells, although this differential expression is lower. Table 1 and supplemental Table S6 [see Additional file 2] list the top genes differentially expressed between siRNA RIP140 RA cells and control siRNA RA cells (Group 4 vs. Group 2). Table 1 ranks the genes by fold-change, while Table S6 [see Additional file 2] ranks the genes by p-value. The known RA-target gene HOXA5 is induced 1.6 fold with RA in siRNA control cells and 20.6-fold with RA in RIP140 siRNA cells for a differential induction of 12.8. RARβ, the primary gene used the establish the negative-feedback model was also identified as being super-induced by RIP140 siRNA (Table 1). Other genes that are highly RIP140-dependent in their RA-alterations include both known (MEIS1, HOXB5, HOXB13) and previously unsuspected (NODAL, TSHZ1) RA target genes. Other noteworthy genes regulated by RA in a RIP140-dependent manner include the nuclear receptor coactivator PCAF and the pluripotency gene FOXD3 which are predicted to be upregulated and downregulated by RA, respectively (Table 1). Including the previously confirmed RARβ gene, we were able to confirm the RIP140-dependent expression pattern of 5 of the top 6 genes from Table 1 (Figure 4).

**Table 1:** Genes differentially expressed with RA in RIP140 siRNA treated NT2/D1 cells

*Gene symbol*	*Gene description*	*RA fold-change in control siRNA cells*	*RA fold-change in RIP140 siRNA cells*	*Differential fold-change*	*p-value*
HOXA5	Homeobox A5	+1.60	+20.66	+12.88	0.000368
TSHZ1	Teashirt family zinc finger 1	+2.15	+18.87	+8.76	0.001052
DMRT1	Doublesex and mab-3 related transcription factor 1	-1.09	+3.99	+4.35	0.010148
C8orf72	Chromosome 8 open reading frame 72	+2.34	+9.85	+4.21	0.022663
ZADH2	Zinc binding alcohol dehydrogenase, domain containing 2	+1.48	+5.67	+3.84	0.011172
VSNL1	Visinin-like 1	-3.89	-14.60	-3.75	0.000767
RARB	Retinoic acid receptor, beta	+7.99	+26.90	+3.37	0.012977
PYCR2	Pyrroline-5-carboxylate reductase family, member 2	+32.66	+104.63	+3.20	0.011152
DEPDC6	DEP domain containing 6	+2.76	+8.67	+3.15	0.026040
KCNJ4	Potassium inwardly-rectifying channel, subfamily J, member 4	+3.72	+11.52	+3.10	0.008146
HOXB5	Homeobox B5	+1.40	+4.19	+2.99	0.031222
MEIS1	Meis1, myeloid ecotropic viral integration site 1 homolog (mouse)	+3.09	+9.06	+2.93	0.022140
-	DKFZP686A01247 hypothetical protein	+1.92	+5.28	+2.76	0.006054
FLJ12624	Phospholipase A2 homolog	-3.20	-8.31	-2.59	0.000778
SUSD3	Sushi domain containing 3	+3.07	+7.89	+2.57	0.023342
EDG7	Endothelial differentiation, lysophosphatidic acid G-protein recept	-1.58	-4.02	-2.55	0.019311
NRP1	Neuropilin 1	+1.54	+3.84	+2.50	0.024092
KIF21B	Kinesin family member 21B	-1.04	+2.37	+2.46	0.001602
INHBB	Inhibin, beta B (activin AB beta polypeptide)	+1.22	+2.95	+2.42	0.001612
HOXB13	Homeobox B13	+1.21	+2.82	+2.34	0.028115
OVOS2	Ovostatin 2	-1.66	-3.85	-2.32	0.001967
HLA-DPB1	Major histocompatibility complex, class II, DP beta 1	-2.07	-4.79	-2.31	0.048107
EMILIN2	Elastin microfibril interfacer 2	-1.65	-3.72	-2.25	0.010781
HAS3	Hyaluronan synthase 3	-1.75	-3.86	-2.21	0.001436
NR6A1	Nuclear receptor subfamily 6, group A, member 1	-1.02	+2.17	+2.21	0.014380
ATBF1	AT motif binding factor 1	+2.93	+6.45	+2.20	0.000101
NODAL	Nodal homolog (mouse)	+1.20	+2.60	+2.16	0.001068
ZNF703	Zinc finger protein 703	+2.62	+5.67	+2.16	0.046510
PCDH17	Protocadherin 17	+2.71	+5.81	+2.14	0.046724
**PAPPA**	**Pregnancy-associated plasma protein A, pappalysin 1**	**+1.43**	**-1.48**	**-2.11**	**0.026579**
KLHL7	Kelch-like 7 (Drosophila)	-1.34	-2.81	-2.09	0.039448
LNX1	Ligand of numb-protein × 1	-1.13	-2.30	-2.04	0.008236
PRKCH	Protein kinase C, eta	+1.99	+4.05	+2.04	0.036242
TBX3	T-box 3 (ulnar mammary syndrome)	+4.45	+9.01	+2.03	0.046092
ADM	Adrenomedullin	-1.15	-2.32	-2.02	0.019731
KCTD12	Potassium channel tetramerisation domain containing 12	-1.03	-2.08	-2.02	0.003013
PTPRU	Protein tyrosine phosphatase, receptor type, U	+1.16	+2.32	+1.99	0.006395
**WNT3**	**Wingless-type MMTV integration site family, member 3**	**+3.05**	**+1.53**	**-1.99**	**0.009256**
MCOLN3	Mucolipin 3	-1.26	-2.48	-1.97	0.041576
PCAF	P300/CBP-associated factor	+1.64	+3.22	+1.97	0.017906
LOC645323	Hypothetical LOC645323	+6.84	+13.40	+1.96	0.002997
EPAS1	Endothelial PAS domain protein 1	+1.01	-1.93	-1.96	0.007540
WDR68	WD repeat domain 68	+1.07	+2.09	+1.96	0.000433
CNR1	Cannabinoid receptor 1 (brain)	+11.84	+23.16	+1.96	0.006641
FAM46B	Family with sequence similarity 46, member B	-3.48	-6.81	-1.95	0.015572
FOXD3	Forkhead box D3	-3.72	-7.26	-1.95	0.018665
PPFIBP2	PTPRF interacting protein, binding protein 2 (liprin beta 2)	-1.17	-2.25	-1.92	0.001765
AK3L1	Adenylate kinase 3-like 1	-4.02	-7.69	-1.91	0.001875
MAP3K5	Mitogen-activated protein kinase kinase kinase 5	-1.12	+1.70	+1.90	0.020647

Gene list represents genes in scatter plot of Figure 3D; Group 4 (siRNA RIP140 RA) vs. Group 2 (control siRNA RA). Genes with a p-value < 0.05 are ranked by fold change between the expression of Group 4 vs. Group 2 (Differential fold change). Values corresponding to scatter plot of Figure 3A, Group 2 vs. Group 1 (RA-fold change in control siRNA cells) and scatter plot of Figure 3C, Group 4 vs. Group 3 (RA-fold change in RIP140 siRNA cells) are also provided. For genes with multiple probe sets, the probe set with the greatest differential expression was chosen. In bold are the two genes where RIP140 siRNA lessens the extent or direction of RA-mediated regulation.

The majority of RIP140-dependent genes change in a manner consistent with RIP140 limiting RA signaling. Hence, RIP140 siRNA results in enhancement or amplification of the RA signal. Genes induced by RA in control cells are induced to a greater extent by RA in RIP140 siRNA cells and genes repressed by RA in control cells are repressed to a greater extent in RIP140 siRNA cells. This trend holds true whether the effects of RIP140 on RA signaling are ranked by fold-change (Table 1, 47 out of 49 genes) or p-value (Table S6, 37 out of 40 genes). The exceptions are bolded in Tables 1 and Table S6 [see Additional file 2]. In addition only 1 gene in the ranked tables had an expression profile that could be consistent with "active repression" (second pattern in Figure 2B). This gene is boxed in Table S6. The effects of RIP140 had little bias for the extent of RA-induction. For example, a strongly induced gene, PYCR2, is induced by RA in control cells 32.6-fold and in RIP140 siRNA cells by 104.6-fold, while a weakly induce gene INHBB is induce 1.22-fold in control cells and 2.95-fold in RIP140 siRNA cells (Table 1). There are also examples of genes that are not altered with RA in control cells but altered by RA in RIP140 siRNA cells. One interpretation of this result is that the RA alteration of these genes may have been accelerated with RIP140 siRNA and that the 24-hour time point is too early to detect RA-mediated changes in the siRNA control cells.

### Silencing of RIP140 does not greatly alter RA-independent gene expression

**Figure 5:**
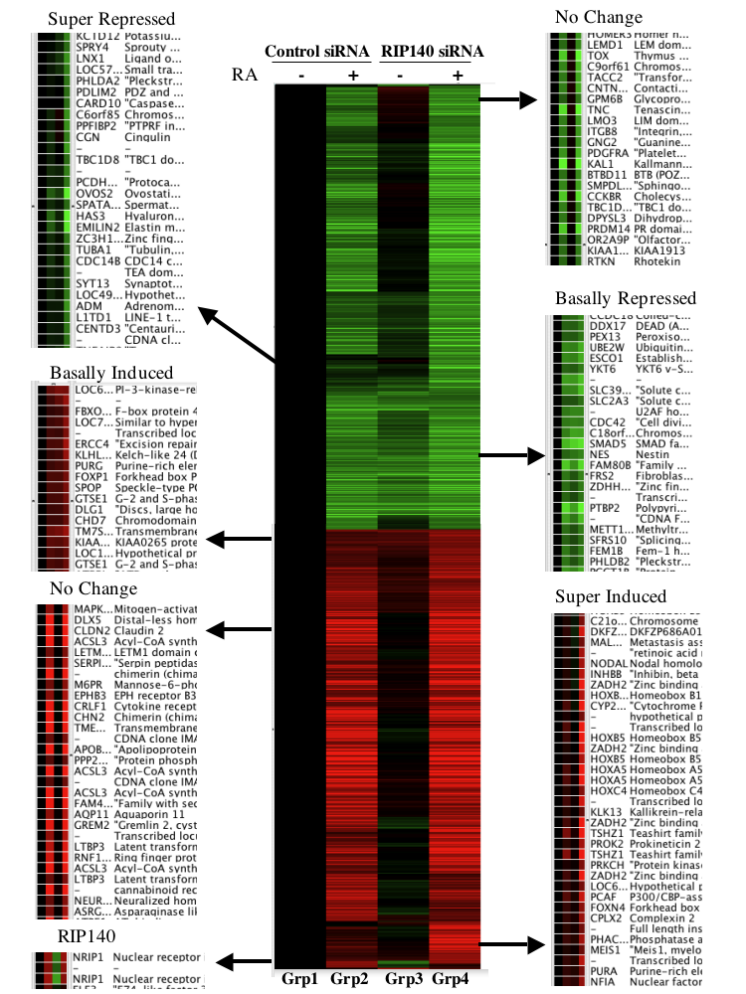
**Hierarchical cluster analysis of expression profile of RA-regulated genes in RIP140 siRNA cells**. The 1115 genes which were changed at least 1.8-fold with p-value < 0.01 in any of the four major groups were hierarchical clustered for similarity. The average expression value of Groups 2, 3 and 4 were compared to the average expression value of Group 1. Red represents genes induced compared to Group 1, green represents genes repressed compared to Group 1. Highlighted clusters are expanded to indicate individual genes and their expression pattern.

Although the array experiments were designed to maximize the potential to find RA-independent RIP140 targets, very few genes matching this pattern were found (last pattern in Figure 2B). This is evident by the comparison of Group 3 vs. Group 1 (Figure 3E, and Table 2 and supplemental Table S7 [see Additional file 3]). Reassuringly, one of the genes identified by the array as repressed basally is RIP140 itself. There is a clear bias for repressed genes suggesting that these genes are indirectly regulated by RIP140. The majority of the altered genes in Figure 3E and Table 2 and Table S7 are also altered with RA in siRNA control and RIP140 siRNA cells (Figure 5). Hence, RIP140 siRNA caused basal alterations in some genes that are altered in control siRNA cells treated with RA. One interpretation of this result is that RIP140 siRNA is sensitizing the cells to the basal levels of retinoids in the normal sera media. Two genes that were found to be exclusively expressed in RIP140 siRNA cells independent of RA, and hence fit the pattern of RA-independent RIP140 targets (last pattern in Figure 2B), were doublecortex (DCX, bolded in Table 2) and reticulon 1 (RTN1, just missed fold-cutoff in Table 2). Both of these genes have roles in neuronal development. Interestingly, a third gene that fit this pattern was only induced 1.47 fold in RIP140 siRNA cells but had a highly significant p-value, the repressor protein, c-terminal binding protein 2 (CTBP2) (Table S7). CTBP2 is a known RIP140 co-factor [20]. This suggests that RIP140 may repress transcription of its own cofactor.

**Table 2:** Genes changed basally with RIP140 siRNA

*Gene symbol*	*Gene description*	*Fold change RIP140 siRNA/ctrl siRNA*	*p-value*
FOLR1	Folate receptor 1 (adult)	-4.65	0.000730
RAB3B	RAB3B, member RAS oncogene family	-4.33	0.005952
SMAD5	SMAD family member 5	-3.28	0.003862
MALAT1	Metastasis associated lung adenocarcinoma 1 (non-coding RNA)	-3.20	0.000468
**DCX**	**Doublecortex; lissencephaly, X-linked (doublecortin)**	**+2.82**	**0.000682**
B3GAT3	Beta-1,3-glucuronyltransferase 3 (glucuronosyltransferase I)	-2.63	0.005142
C14orf111	Chromosome 14 open reading frame 111	-2.62	0.003088
SMAD5	SMAD family member 5	-2.60	0.006314
CUTL1	Cut-like 1, CCAAT displacement protein (Drosophila)	-2.52	0.001457
C18orf54	Chromosome 18 open reading frame 54	-2.51	0.003054
PTBP2	Polypyrimidine tract binding protein 2	-2.48	0.004811
**NRIP1**	**Nuclear receptor interacting protein 1**	**-2.42**	**0.011665**
RSN	Restin (Reed-Steinberg expressed intermediate filament-associated)	-2.36	0.007189
UHMK1	U2AF homology motif (UHM) kinase 1	-2.34	0.009057
FNIP1	Folliculin interacting protein 1	-2.34	0.003628
PCBP2	Poly(rC) binding protein 2	-2.28	0.001479
BAT2D1	BAT2 domain containing 1	-2.28	0.001042
FAM80B	Family with sequence similarity 80, member B	-2.26	0.008836
UHMK1	U2AF homology motif (UHM) kinase 1	-2.26	0.002063
XKR6	XK, Kell blood group complex subunit-related family, member 6	-2.23	0.001595
BACH1	BTB and CNC homology 1, basic leucine zip transcription factor 1	-2.22	0.006338
LOC158863	Hypothetical protein LOC158863	-2.22	0.001177
GPD2	Glycerol-3-phosphate dehydrogenase 2 (mitochondrial)	-2.22	0.004723
LOC552889	Hypothetical LOC552889	-2.18	0.014483
RBAK	RB-associated KRAB zinc finger	-2.17	0.001749
ZFR	Zinc finger RNA binding protein	-2.14	0.001579
SLC39A14	Solute carrier family 39 (zinc transporter), member 14	-2.14	0.000483
UHMK1	U2AF homology motif (UHM) kinase 1	-2.12	0.000315
HNRPU	Heterogen nuclear ribonucleoprotein U (scaffold attach factor A)	-2.12	0.000802
PGGT1B	Protein geranylgeranyltransferase type I, beta subunit	-2.10	0.002873
ZNF660	Zinc finger protein 660	-2.09	0.001916
ZNF655	Zinc finger protein 655	-2.09	0.011026
CDC42	Cell division cycle 42 (GTP binding protein, 25 kDa)	-2.08	0.001266
ESCO1	Establishment of cohesion 1 homolog 1 (S. cerevisiae)	-2.08	0.002092
UBE2W	Ubiquitin-conjugating enzyme E2W (putative)	-2.07	0.010394
HECTD1	HECT domain containing 1	-2.06	0.012537
UFM1	Ubiquitin-fold modifier 1	-2.05	0.003571
LOC641298	PI-3-kinase-related kinase SMG-1 – like locus	+2.03	0.000555
GCNT2	Glucosaminyl (N-acetyl) transferase 2, (I blood group)	-2.03	0.001021
EDG7	Endothelial differentiation, lysophosphatidic acid G-coupled recept	-2.03	0.013785

Gene list represents genes in scatter plot of Figure 3E; Group 3 (siRNA RIP140 vehicle) vs. Group 1 (control siRNA vehicle). Genes with p-value < 0.05 are ranked by fold-change. In bold is DCX the one gene changed basally in siRNA RIP140 cells that is not also changed by RA in control siRNA cells. Also in bold is RIP140 (NRIP1). For genes with multiple probe sets, the probe set with the greatest differential expression was chosen.

### RA target genes cluster into distinct groups based on RIP140-dependency

**Figure 6:**
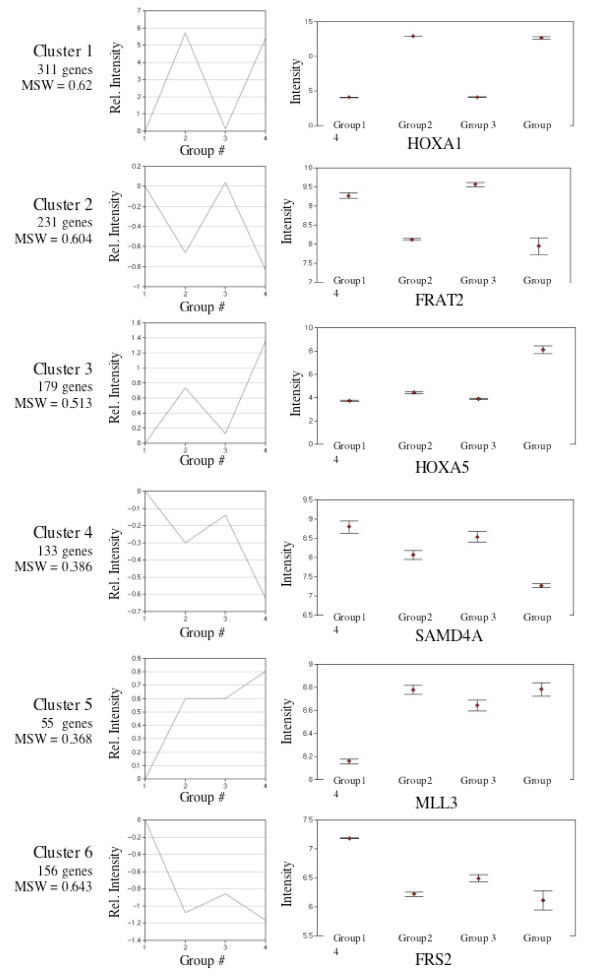
**Partitioning around medoids (PAM) analysis of RA-regulated genes**. The 1065 genes changed 1.5-fold or greater with a p-value of < 0.001 were subjected to PAM analysis utilizing GeneSifter software as described in Methods. The number of genes in each of the six clusters and the mean silhouette width (MSW) value for each cluster is indicated. Group 1 is control siRNA vehicle. Group 2 is control siRNA RA. Group 3 is RIP140 siRNA vehicle. Group 4 is RIP140 siRNA RA. Expression intensity values for a representative gene in each group is provided on left. Error bars are S.E.M.

**Figure 7:**
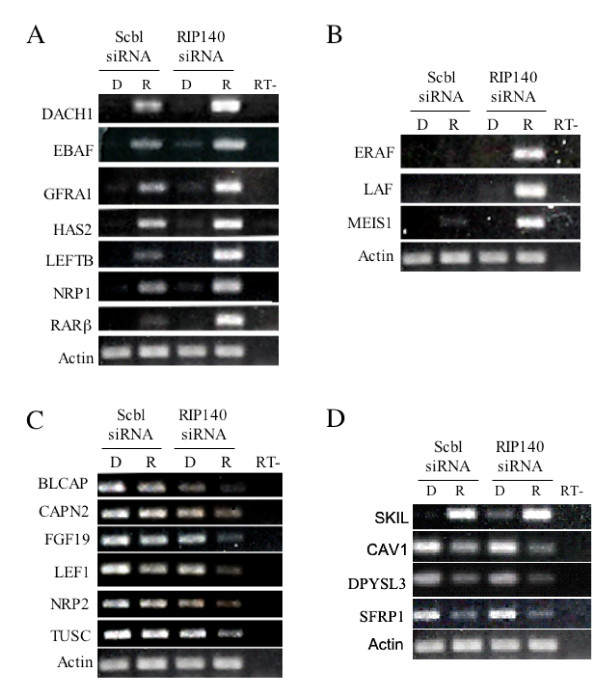
**RIP140 siRNA effects RA target expression**. RT-PCR analysis of NT2/D1 cells transfected with scrambled siRNA or RIP140 siRNA#1 and treated with DMSO (D) or 10 μM RA (R) for 24 hours. These samples are independent of those used for microaray analysis. Genes were selected from array analysis or are known RA target genes. In indicated samples (-RT) reverse transcriptase was omitted to control for genomic DNA contamination. Actin control is repeated for each figure for clarity. Pattern of genes for each figure are, super-induced with RIP140 siRNA **(A and B)**, super-repressed with RIP140 siRNA **(C)**, and no change with RIP140 siRNA **(D)**.

The major influences of RIP140 siRNA were also assessed by hierarchical clustering. There were 1,115 genes with a fold change of 1.8 or greater in any of the four groups with p-value < 0.01 (supplied as Supplemental Table S8 [see Additional file 4]). These were clustered by the Eisen program (Figure 5). Inspection of the cluster confirms that the major pattern of RIP140 siRNA alterations is RA-dependent. In that, very few genes made to threshold cutoffs because of RA-independent alterations in expression (only changed in Group 3 and Group 4 of Figure 5). Further, the pattern associated with active repression (Figure 2B) was not greatly evident (would be green in Group 2 but less green in Group 4 of Figure 5). Genes representative of the major patterns are shown in Figure 5. These include genes that are basally induced or repressed with RIP140 siRNA, and genes that are "super-induced" or "super-repressed" by RIP140 siRNA. Approximately half of the total RA-dependent genes were not influenced by RIP140 in this analysis. Partitioning around mediods (PAM) analysis was also performed on genes with a cut off of 1.5-fold and p-value of < .001, (1065 genes) (Figure 6). There were 542 genes in cluster patterns indicative of little or no change with RIP140 siRNA (Cluster 1 and Cluster 2). There were 312 genes in cluster patterns indicative of super-induction or super-repression with RIP140 siRNA (Cluster 3 and Cluster 4) and 211 genes in clusters indicative of basal induction or basal repression in RIP140 siRNA cells (Cluster 5 and Cluster 6, Figure 6). The list of genes and expression values for each of the six clusters is supplied as Supplementary Tables S9 to S14 [see Additional file 5]. Genes representative of some of the patterns were confirmed in independent samples (Figure 7).

### Mode of action of RIP140 in RA signaling

**Figure 8:**
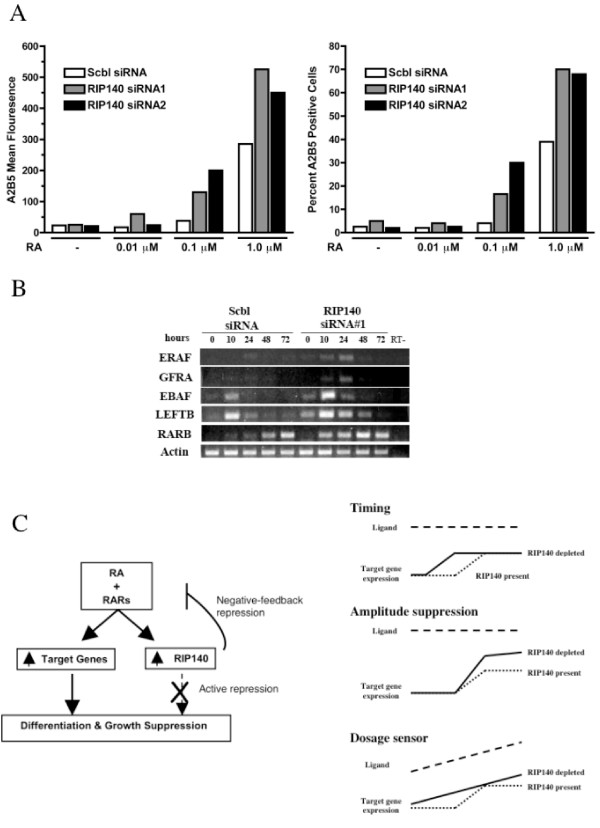
**RIP140 silencing sensitizes NT2/D1 cells to low dose RA and accelerates and amplifies RA-dependent expression of RA-target genes**. **(A) **Immunophenotypic FACS analysis for the established neuroectodermal marker A2B5 in NT2/D1 cells transfected with 40 nM scrambled siRNA (Scbl) or RIP140 siRNA#1 or RIP140 siRNA#2 and treated for 4 days with DMSO vehicle control or indicted dosages of RA. Data are presented as mean fluorescence **(left) **and percent positive cells **(right)**. **(B) **RT-PCR analysis of NT2/D1 cells transfected with 60 nM scrambled siRNA (Scbl) or RIP140 siRNA#1 and treated with 10 μM RA for the indicated time points. Representative of two experiments. **(C) **Models of RIP140 regulation of RA signaling supported by the current study. See text for details.

The pattern of gene expression in RIP140 siRNA cells suggests that RIP140 siRNA may influence RA signaling in distinct manners, either to sensitize cells to lowdose RA (basally altered array genes) or to accelerate or amplify RA-dependent gene expression (super-induced or super-repressed array genes). However, the array was performed at a single RA dose and time point. We had shown previously that RIP140 siRNA accelerates and enhances RA-induced differentiation in NT2/D1 cells [22]. RIP140 siRNA was also able to promote differentiation of NT2/D1 cells at low doses of RA (Figure 8A). Time course studies demonstrated that RIP140 siRNA caused a combination of accelerated and amplified alterations in RA target gene expression (Figure 8B). Hence, these data suggests that RIP140 may limit a subset of RA target genes by effecting the rate and extent of target gene expression at a given dose of RA.

## Discussion

Through the use of microarray technology we sought to elucidate, in a *de novo *fashion, the global role of RIP140 in RA-dependent signaling. The predominant gene expression pattern is consistent with RIP140 limiting RA signaling [22]. Since RIP140 depletion enhances RA-induced differentiation (Figure 1B, 8A) these genes may play an important role in mediating the growth suppression, differentiation and anti-tumorigenic effect of RA in embryonal carcinoma. These data support the notion that the primary effect of the ligand-dependent corepressor RIP140 is to limit the activity of retinoid receptors (Figure 8C). A feature of this model is that RIP140 is itself induced by RA [21] and that RIP140 inhibits ligand-bound RARs to form a classic negative-feedback loop. One interesting gene predicted to be repressed by RIP140 was the RIP140 cofactor, CTBP2 [20]. This raises the potential of the additional complexity of RIP140 limiting its own activity by regulating CTBP2. Confirming and establishing the importance of RIP140 transcriptional regulation of CTBP2 will require further rigorous studies.

It is tempting to speculate on the importance of RIP140 restriction of RA-target gene expression. One possibility is that RIP140 ensures that cells are exposed to a threshold concentration of RA for a required duration before committing to differentiation. This could be important for developmental timing but an impediment to retinoid therapies in the adult [1,25]. Alternatively, RIP40 may fine-tune the RA signal to ensure that a critical set of target genes are not overly or toxically expressed.

Our studies suggest that regulation of RAR signaling by RIP140 is more complex than a simple all-or-none effect on global RAR activation. We have identified gene subsets regulated by RA in apparent RIP140-dependent and RIP140-independent manners. It is possible that RIP140 may have a selective ability to affect the actions of different subtypes of retinoid receptors. Alternatively, this differential effect of RIP140 may involve differences in the local chromatin environment of specific genes. First-pass analysis of the predicted promoter regions of RIP140-dependent target genes was unable to identify any *cis*-acting features that may account for differential sensitivity to RIP140. We believe the RIP140-sensitive RA target genes identified here may have pharmacologic importance during normal retinoid-based cancer therapy or prevention [1,25]. It would also be interesting to know the number of RIP140 sensitive genes that are direct RA-target genes.

Within the limits of the single RA dose and time-point employed, RIP140-dependent genes can be placed in three categories based on their expression profiles (Figure 5, 6 and 7). Some genes appeared super-induced or super-repressed with RIP140 repression, meaning they were changed with RA to a greater degree in RIP140 siRNA cells compared to control siRNA cells. Some genes were induced with RA in RIP140 siRNA cells but unchanged by RA in control siRNA cells. It may be predicted that this subset of genes is regulated by RA in the control siRNA cells at later time points. Some RA regulated genes in control cells were regulated basally in the siRNA RIP140 cells. We propose that these genes are expressed in RIP140 siRNA cells due to the presence of previously sub-optimal concentrations of retinoids in standard non-charcoal stripped FBS media. Hence, from the data presented here we propose the RIP140 plays a role in finetuning the RA signal by either delaying the timing, decreasing the amplitude or decreasing the effective ligand concentration of RAR activation (Figure 8C).

Recent microarray studies suggest extensive and early ligand-dependent repression of gene expression by nuclear receptors [12,13]. However, the mechanism for such repression is unclear. Ligand-dependent repressors like RIP140 are candidate mediators of such repression. This is particularly relevant for the steroid nuclear receptors such as the estrogen, progesterone and glucocorticoid receptors. If RIP140 participates in this form of RAR "active repression" one would expect that genes repressed by RA would be de-repressed upon RIP140 silencing. Our data does not support a widespread role for RIP140 in retinoid receptor-mediated active repression. This conclusion is consistent with the finding that RIP140 also inhibits glucocorticoid receptor-mediated repression from a negative GRE [26]. As with other nuclear receptor and coregulator functions, active repression by RIP140 may be cell-, receptor- and context-specific.

While it is known that RIP140 modulates transcription through repressing ligand-dependent nuclear receptors, the possibility exists that RIP140 may function through multiple mechanisms, including nuclear receptor-independent mechanisms. For example, a recent microarray study reported a role for the coactivator, NCoA3/AIB1, in receptor-independent activities, illustrating the diverse functionality of coregulators and highlighting the need for further elucidation of their role in gene transcription [27]. The microarray studies presented here were optimized to investigate gene changes following RA treatment, making it difficult to draw conclusions regarding the effects of RIP140 on other nuclear receptors. Despite the inclusion of normal sera media which may contain limiting quantities of ligand for other nuclear receptors, we were unable to identify many RA-independent targets of RIP140. To address this question, additional RIP140 siRNA array studies should be performed with exogenous ligands for other nuclear receptors.

A limited number of studies have been performed to assess the global effect of RIP140 on gene expression. Expression profiles were performed with murine embryonic fibroblasts in which RIP140 was deleted and reintroduced during adipocytic differentiation [28]. Another study employed 3T3-L1 fibroblasts treated with RIP140 siRNA during adipocytic differentiation [29]. Genes identified to be regulated by RIP140 in these systems were involved in glucose and fatty acid metabolism [28,29]. An additional study profiled mouse ovaries from RIP140 knockout mice treated with or without human chorionic gonadotropin and pregnant mare serum gonadotropin [30]. There is little overlap in the RIP140-dependent genes identified in these and our study. This is not surprising since RIP140 likely regulates different sets of genes in different tissues based on the ligand/receptor under investigation and the distinct hormone sensitivities of the tissue.

## Conclusion

The data demonstrates that RIP140 globally effects RA-mediated gene expression and that RIP140 discriminates between different classes of RA targets in the embryonal carcinoma cancer stem cell model. Maintaining therapeutic doses of RA has proven difficult in the clinic [1,25]. Understanding the mechanisms that limit RA effectiveness may aid in developing new RA therapeutic strategies. We propose that the combination of RIP140 gene silencing with RA treatment might enhance expression of important RA target genes, thereby enhancing the therapeutic benefit of RA. Future studies should focus on pharmacologic modulation of RIP140 expression or activity as a potential strategy to enhance the chemotherapeutic action of retinoids.

## Methods

### Cell culture, siRNA and transfection protocols

The RA-sensitive human EC cell line, NT2/D1 is a clonal line derived from a xenograft of Tera-2 cells [31]. Cells were cultured in high glucose DMEM (Gibco) with 10% FBS supplemented with glutamine and antibiotics. Repression of RIP140 was achieved using three siRNAs purchased from Dharmacon. RIP140siRNA#1 and RIP140siRNA#2 have been previously described [22,23]. RIP140siRNA#1 is of sequence 5'-GAAGGAAGCUUUGCUAGCU-3' and corresponds to the human RIP140 cDNA starting 331 bp downstream of the ATG start codon. RIP140siRNA#2 is of sequence 5'-CAAACAGGAUAGCACAUUA-3' and corresponds to human RIP140 cDNA starting 1986 bp downstream of the ATG start codon. RIP140siRNA#3 is of sequence 5'-AACUGUGAGUAGCCUAUGA-3' and corresponds to the 3'-UTR of RIP140 242 bp downstream of the stop codon. Controls were the Scramble II siRNA or RISC-free siRNA from Dharmacon. For microarray experiments, log-phase cells were transfected with 90 nM siRNA using OligofectAMINE (Invitrogen) as described [22,23]. Briefly, 3.5 × 10^6 ^cells per 10 cm plate were transfected with 90 nM siRNA for 16 h. 1 × 10^6 ^cells were then replated in normal sera DMEM media per 10 cm dish and 24 hours later treated with either 10 μM RA or DMSO for 24 hours before harvesting for mRNA. For some indicated experiments, NT2/D1 cells were first incubated in charcoal-absorbed sera containing DMEM prior to transfection with siRNA and treatment with indicated dosages of RA.

### Reporter assays

The reporter, RARE-TK-Luc, and the expression plasmid pSG5-HAhRIP140 have been previously described [21-23]. Cells were plated at 1.5 × 10^5 ^cells per well of a six-well plate and transfected with Polyfect (Qiagen) according to the manufacturer's directions. Tranfections were performed in triplicate for each condition with a final concentration of 0.375 μg RARE-TK-Luc, 0.375 μg pRL-TK, 0.75 μg of either pSG5-insertless or pSG5HA-hRIP140, and 50 nM of control or RIP140 siRNA. Cells were exposed to transfection reagent for 16 hours and then washed and cultured in indicated dosages of RA or the vehicle, DMSO. Cells were harvested 48 hours following transfection and Dual Luciferase Reporter analysis was performed (Promega). Luciferase activity was normalized against renilla activity and reported as the average of triplicate transfections with similar results obtained in at least two independent experiments.

### Northern and RT-PCR analysis

Total RNA was harvested using TriReagent (Invitrogen). Northern hybridizations for RARβ and RIP140 were performed with 5 μg RNA as previously described [22]. Expression levels of RA-target genes were assessed by semi-quantitative RT-PCR. The cDNA was synthesized using Superscript II RT from 5 μg total RNA. PCR analysis was performed with *Taq *polymerase (Denville) to varying cycle numbers within the linear range as previously described [22]. The sequence of primers are available upon request.

### Immunophenotypic analyses

Indirect fluorescence activated cell sorter (FACS) analysis to evaluate RA-induced neuronal differentiation of NT2/D1 cells was performed using established techniques [32]. Briefly, NT2/D1 cells were harvested by trypsinization and incubated with antibody to the cell surface antigen A2B5 or with isotype-matched monoclonal antibody as controls. The A2B5 antibody recognizes a neuronal epitope on RA-treated NT2/D1 cells and was prepared from hybridoma cultures purchased from ATCC. The cells were indirectly assayed with FITC-conjugated goat-anti-mouse antibody, and fluorescence was measured as described [32]. Mean peak fluorescent values and the percentage of positive cells were measured for the entire population. At least 1 × 10^4 ^cells were analyzed per assay.

### Microarray analysis

Total RNA was purified using RNeasy columns (Qiagen). Hybridizations were performed according to Affymetrix guidelines at the Dartmouth College Microarray Shared Resource using an Affymetrix GeneChip Workstation. Biotin-labeled cRNA was generated from 5 μg of total RNA and hybridized to the Human Genome (HG) U133 Plus 2.0 chip which contains over 54,000 probe sets. Unless specifically stated we will refer to each probe set as a "gene". As described in Figure 2A, a total of 12 hybridizations were performed comprising 12-independent biologic samples organized into four groups of three. This data is available at the GEO website under Accession GSE7500 and conforms to MIAME guidelines. Raw data from each hybridization was normalized by RMA, background corrected and filtered for present using GeneSifter software (vizX Labs). As a further filter, any gene that showed a 1.5-fold or greater difference amongst the three samples comprising Group 1 (the siRNA controls) were eliminated from further analysis. The remaining 52,270 probe sets were then analyzed by GeneSifter software. Pairwise comparisons between groups were performed using t-test with p-value and cutoffs as indicated. For hierarchical cluster analysis genes changed 1.8 fold or greater between any of the four groups with p-value < .01 (1115 genes) were clustered via the Cluster program by Michael Eisen using a correlation metric for similarity matrix and average linkage clustering [33]. The clustered data was visualized by Tree-View software. Genes were also divided into clusters using partitioning around medoids (PAM) analysis from the GeneSifter package. Genes changed 1.5-fold or greater with a p-value of < .001 (1065 genes) were used for this analysis using the correlation metric for similarity. The number of clusters (six) was chosen empirically to obtain the best mean silhouette value of 0.560.

## Abbreviations used

NRIP1, nuclear receptor interacting protein 1; RA, retinoic acid; EC, embryonal carcinoma; RAR, retinoic acid receptor; RXR, retinoid X receptor; NcoR, nuclear receptor co-repressor 1; SMRT, silencing mediator for retinoid and thyroid hormone receptor; CTBP, c-terminal binding protein.

## Competing interests

The authors declare that they have no competing interests.

## Authors' contributions

KCH carried out sample characterization for the reported arrays, participated in the array experiments and final data analysis, performed Northern and RT-PCR analysis. KAW-B participated in the initial study design, preliminary array experiments and data interpretation, expression confirmations and FACs analysis. DD participated in reporter experiments, RT-PCR, FACS and Northern analysis. CRT directed and supervised microarray studies. JHM participated in extraction and analysis of gene expression data from the microarray. SJF contributed to RNA sample preparation and study design. MJS conceive of the study, directed the study and participated in the extraction and analysis of gene expression data, data interpretation and writing of the manuscript. All authors read and approved the final manuscript.

## Supplementary Material

Additional file 1 Supplemental Tables 1 through 5. Complete list of genes and expression values for each pairwise comparison in Figure 3. Click here for file

Additional file 2 Supplemental Table 6. Top genes differentially expressed in siRNA RIP140 RA cells versus control siRNA RA cells ranked by p-value. Click here for file

Additional file 3 Supplemental Table 7. Top genes changed basally with RIP140 siRNA ranked by p-value. Click here for file

Additional file 4 Supplemental Table 8. The 1,115 genes in Figure 5 with a fold-change of 1.8 or greater in any of the four treatment groups with p-value < 0.01. Click here for file

Additional file 5 Supplemental Tables 9 through 14. The list of genes and expression values for each of the six clusters in Figure 6. Click here for file
